# Association between indoor ventilation frequency and frailty among Chinese older adults

**DOI:** 10.3389/fpubh.2025.1670577

**Published:** 2025-12-01

**Authors:** Xiaobing Xian, Xiyu Chen, Qian Wang, Shiwei Cao, Heqian Fan, Li Zeng, Qiwei Tang, Luxi Chen, Yuanyuan Wang, Kun Shen

**Affiliations:** 1 The Thirteenth People’s Hospital of Chongqing, Chongqing, China; 2 Chongqing Geriatrics Hospital, Chongqing, China; 3 The First Clinical College, Chongqing Medical University, Chongqing, China; 4 The Second Clinical College, Chongqing Medical University, Chongqing, China; 5 Department of Pediatrics, Chongqing Medical University, Chongqing, China; 6 School of Mathematics and Statistics, Chongqing Technology and Business University, Chongqing, China

**Keywords:** frailty, indoor ventilation frequency, older adults, CLHLS, external validation

## Abstract

**Background:**

Frailty is a common geriatric syndrome that imposes a heavy disease burden globally. Indoor ventilation is a crucial measure for improving air quality. However, the association between indoor ventilation frequency (IVF) and frailty remains unclear. This study aimed to explore this association among Chinese older adults.

**Methods:**

We used data from 5,511 older adults in the Chinese Longitudinal Healthy Longevity Survey (CLHLS) 2018 and an external validation sample of 718 older adults from Chongqing. Logistic regression models and linear regression models were employed to assess the association between IVF and frailty and its seasonal variations. We further conducted subgroup analysis to examine differences across various populations. All statistical analyses were performed using SPSS 25.0 and R 4.3.0.

**Results:**

Compared with low ventilation frequency, intermediate (OR = 0.722, 95% CI: 0.559 ~ 0.933) and high (OR = 0.824, 95% CI: 0.643 ~ 0.995) frequencies were significantly associated with a lower risk of frailty. Seasonal analysis revealed that this inverse association was particularly significant in autumn and winter. Subgroup analysis suggested that this association was more pronounced in subgroups such as females, older adults over 80 years old, and those who use non-clear energy for cooking. External validation data from Chongqing supported these findings.

**Conclusion:**

This research demonstrated a significant association between IVF and frailty among Chinese older adults. These findings provide supportive evidence for considering ventilation behavior in public health strategies aimed at promoting healthy aging.

## 1 Introduction

Frailty is a prevalent clinical syndrome in geriatric medicine research, typically accompanied by weight loss, decreased grip strength, slow walking speed, reduced physical function, and limited physical activity (1–3). As the global population ages, the prevalence of frail older adults is escalating annually, posing an emerging global health burden (1, 4). A global systematic review and meta-analysis about frailty in 2021 showed that the prevalence of frailty among middle-aged and older adults over 50 years old has reached about 7% (5). What’s more, a recent 2023 study found that in Japan, a country with a severely aging population after the COVID-19 pandemic, the incidence of frailty among individuals aged 70–75 has risen to 17.4%, bringing significant pressure on medical and nursing institutions (6). Due to the decline in physiological reserves of multiple systems (e.g., nervous, muscular, metabolic, immune), and a reduced ability to withstand stressors, older adults with frailty are more susceptible to adverse clinical outcomes, including increased complications, accidental injuries (such as falls or fractures), disability, poor quality of life (7–10). This increase in care needs places a substantial burden on healthcare systems and society. However, most healthcare systems are inadequately prepared and lack sufficient resources to manage the chronic and complex needs of a large vulnerable older adults, creating significant challenges for clinical practice and public health (11). Therefore, it is imperative to identify modifiable factors associated with frailty and explore strategies to mitigate its progression to promote healthy aging.

With rapid technological and economic development, and a growing public demand for a higher quality of life, scholars have increasingly focused on indoor air pollution (12–14). According to a World Health Organization report from 2019, indoor air pollution is currently five to 10 times more common than outdoor pollution. It is also one of the top 10 health threats to people worldwide, with nearly half of the world’s population being exposed to it. Previous studies have shown that indoor air pollutants, such as particulate matter (PM_2.5_) and volatile organic compounds (like formaldehyde), pose serious health risks and have been linked to adverse outcomes, including cancer, leukemia, and miscarriage (14–16). Furthermore, a study published in 2021 estimated that poor indoor air quality causes more than 5 million premature deaths globally each year (15). This also results in millions of losses due to reduced employee productivity, material losses, and increased healthcare costs, further emphasizing indoor air pollution’s significant impact on patients and even the utilization of social resources.

More and more studies have found that indoor ventilation, which improves air quality, is directly relevant to the health of older adults in recent years (17). However, most related research has focused on methods to improve air quality (14, 15, 18). Studies on ventilation have primarily examined the relationship between indoor ventilation frequency (IVF) and the health of specific populations (such as subway passengers or workers) in certain scenarios (19, 20), rather than the association between IVF and the common geriatric syndromes in the general older adults. To our knowledge, no epidemiological study to date has investigated the association between IVF and frailty among community-dwelling older adults.

Based on the cross-sectional data from China Longevity and Health Longitudinal Survey (CLHLS) and a validation survey from Chongqing, this study aims to explore the relationship between IVF and frailty among Chinese older adults. It offers a way to translate existing data resources into clinical practice and public health strategies, providing a reference for healthcare policy formulation aimed at healthy aging.

## 2 Materials and methods

### 2.1 Data source and participants

Data from the 2018 wave of the CLHLS were used in this study. This ongoing longitudinal study collected information from adults over 60 on basic status, income sources, health and quality of life, personality and psychological features, and other topics. Participants of this study, who made up approximately 85% of China’s total population, resided in 23 different provinces around China. All participants provided written informed consent, and the study was approved by Peking University Biomedical Ethics Committee (IRB00001052-13074).

**Figure 1:**
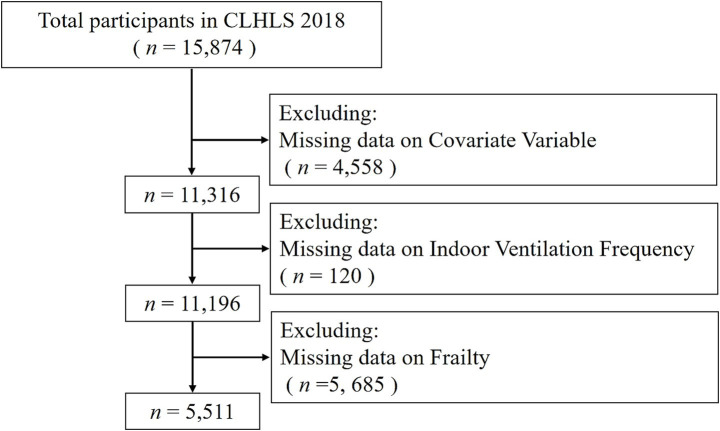
Flowchart showing the selection of the participants enrolled in the CLHLS.

**Figure 2:**
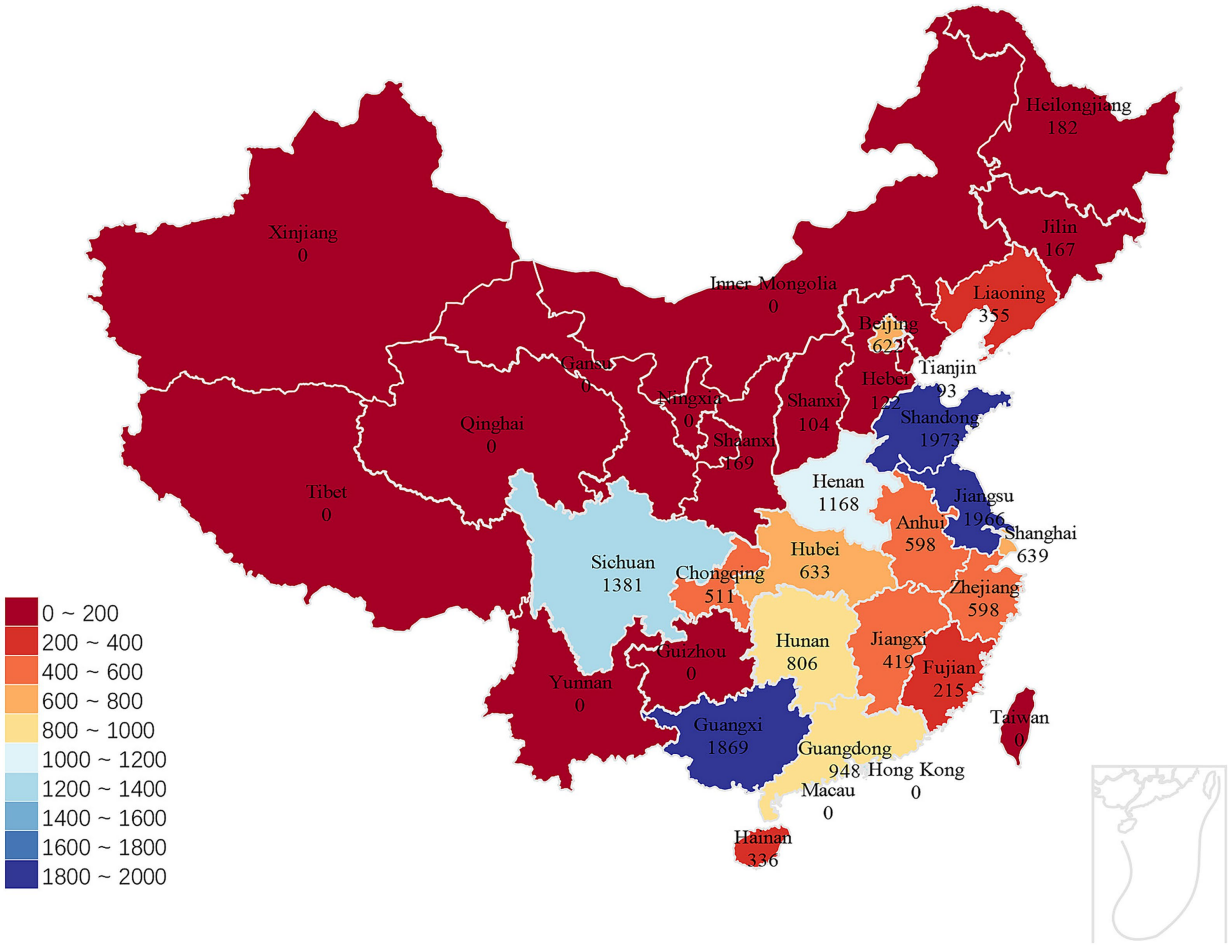
Geographical distribution of the study participants across China. Created using data from “The Chinese Longitudinal Healthy Longevity Survey (CLHLS)-Longitudinal Data (1998-2018)”, map created using Microsoft Excel.

The sample size was estimated using a standard formula for cross-sectional studies [ $N=(Z_{\alpha /2}^{2}p(1-p))/\delta^{2}$ ], where *N* is the required sample size, *Z*_α/2_ is the critical value (1.96) for a two-tailed test at *α* = 0.05, P is the anticipated prevalence of frailty, and δ is the margin of error (0.1P). Considering that the prevalence of frailty is 32.3% older adults over 65 in China (21), about 410 participants were needed for this study to meet its sample size requirements. The final analytical sample size far exceeded this minimum requirement, ensuring sufficient statistical power. A total of 15,874 individuals participated in the 2018 CLHLS survey. We excluded 4,558 participants with missing data on any of the 20 covariates, 120 with missing IVF data, and 5,685 with missing frailty data. The final analytical sample comprised 5,511 participants, and the selection process is detailed in Figure 1. The geographical distribution of participants is shown in Figure 2, which uses a color gradient to represent sample size density across China’s provincial administrative divisions. The study subjects of this research are widely distributed in the eastern and central regions of China, showing good geographical diversity and national representativeness.

Additionally, we conducted an independent cross-sectional survey in Chongqing, China, for external validation of the association between IVF and frailty. From June to July 2025, we enrolled 718 participants among the elderly patients (aged ≥ 65 years) from the Thirteenth People’s Hospital of Chongqing (Chongqing Geriatric Hospital). According to medical records, participants were recruited from various communities in Chongqing’s main urban area. Frailty was assessed using the same criteria as in the CLHLS analysis. Trained investigators conducted face-to-face interviews using a structured questionnaire. This survey was conducted in accordance with the Declaration of Helsinki and approved by the Ethics Committee of the Thirteenth People’s Hospital of Chongqing: No. 2025-022-01 (Research Ethics Approval). All participants provided written informed consent after being fully informed about the study’s purpose and procedures.

### 2.2 Covariate variable

We considered 20 covariates encompassing sociodemographic characteristics, lifestyle, social participation, and air pollution exposure as potential confounders. Sociodemographic factors included age, gender, residence, living arrangement, education, and marital status. Lifestyle factors included smoking, drinking, and exercise. In addition, four variables related to outdoor pollution, namely mold exposure, distance of residence from the main road, kitchen’s ventilation status, and method for cooking, were also taken into account. Social participation was a binary categorical variable assessed by the overall score about the frequency of Taichi, square dancing, gardening, reading, feeding pets, playing cards, watching TV or video, and other social activities. Each activity was scored from 1 (“never”) to 3 (“almost every day”). The scores were summed, and a total score <14 was defined as poor social participation (coded as 0), while a score ≥14 was defined as good social participation (coded as 1).

As a high-quality database with high spatial resolution and long-time series, the China High Air Pollution (CHAP) dataset integrates multi-source data such as ground monitoring station data, satellite remote-sensing data, and meteorological model simulation data through complex machine-learning algorithms to generate pollutant concentrations covering different regions across the country, showing high accuracy^1^. Regional concentration data are mainly obtained through a land-use regression model with corrected time and cloud cover in urban areas, while the concentrations are adjusted using ozone monitoring instrument satellite data in rural areas. The Spatio-Temporal Extreme Randomized Trees (STET) model was used to estimate the daily pollutant concentrations (22). We precisely matched the annual average concentrations of daily outdoor pollutants across different regions in 2018 (fully consistent with the CLHLS survey year) from the CHAP database to each respondent using provincial administrative codes embedded in participant IDs. All provinces where respondents resided had complete air pollution concentration data. Six regional pollution indicators, including carbon monoxide (CO), nitrogen dioxide (NO_2_), ozone (O_3_), particulate matter with an aerodynamic diameter ≤ 2.5 μm (PM_2.5_), particulate matter with an aerodynamic diameter ≤ 10 μm (PM_10_), and sulfur dioxide (SO_2_), were incorporated into the model. Using quartiles as cut-off points, these variables were categorized into four groups for model inclusion. Detailed information is provided in Supplementary Table 1.

### 2.3 Indoor ventilation frequency

IVF was assessed based on self-reported window-opening frequency during four seasons. Considering that in large-scale epidemiological studies, conducting detailed real-time measurements of ventilation requires substantial human resources and economic costs, making it less feasible. This self-reported habitual frequency has been widely validated as a reliable and practical proxy indicator reflecting long-term ventilation behavior and has been confirmed to be associated with various health outcomes in previous studies (23, 24). We believe that this measurement method is reasonable. Participants were asked, “How often do you open your windows each week in each season?” The response options included: never open windows, 1–3 times per week, 3–5 times per week, and > 5 times per week. The responses were uniformly coded: 0 was assigned for never opening windows, 1 for 1–5 times per week, and 2 for > 5 times per week.

We summed the indoor ventilation scores of the four seasons to obtain an overall cumulative IVF score ranging from 0 to 8. This score is intended to comprehensively reflect an individual’s continuous ventilation habits throughout the year, which can better represent long-term exposure levels than single-season measurements. Lowess smooth plots were employed to assess the association between continuous IVF and frailty, with the optimal smoothing parameter (bandwidth = 0.75) determined via 5-fold cross-validation. The analysis revealed a nonlinear negative correlation between cumulative IVF scores and frailty risk (Supplementary Figure 1). A distinct inflection point was observed near IVF total scores of approximately 4 and 5, indicating a critical threshold for trend transition in the association. Combining the results of the Lowess curve with previous authoritative literature based on the CLHLS database (23, 24), we divided the cumulative scores into three levels: low (0–3 points), medium (4–5 points), and high (6–8 points). This classification method is generally consistent with the trend revealed by the Lowess curve and can capture the health differences among people with different ventilation levels.

### 2.4 Frailty

We constructed a frailty index to measure and assess an individual’s vulnerability based on methods used in previous CLHLS studies (25, 26). This 39-item measure covers instrumental activities of daily living with functional limitations, cognitive function, self-reported and interviewer-assessed health, mental health, sensory function (hearing, vision), and the presence and impact of chronic diseases. Thirty-eight items were scored as 0 (deficit absent) or 1 (deficit present). For one item pertaining to severe disorders (e.g., stroke, cancer, and cataracts) that resulted in hospitalization and being bedridden in the past 2 years, a score of 2 was assigned if two or more such conditions were reported. The frailty index was calculated as the sum of all deficit scores divided by the total number of deficits considered, resulting in a continuous score ranging from 0 to 1. Following established cut-offs (27, 28), participants were categorized as non-frailty (≤0.21) and frailty (>0.21). A higher FI score indicates a greater degree of frailty. The specific items and coding details are provided in Supplementary Table 2.

### 2.5 Statistical analysis

In the final dataset without any missing values, the Kolmogorov–Smirnov test was employed to examine the normality of continuous variables. Non-normally distributed continuous variables are presented as median with interquartile range [M (Q1, Q3)], and were compared using the Mann–Whitney *U* or the Kruskal–Wallis test. Categorical variables are expressed as frequencies and percentages [*n* (%)], and group differences were analyzed using the Chi-square test or Fisher’s exact test (when expected cell frequencies were <1 or total *n* < 40).

Three regression models were constructed to examine the association between IVF and frailty. Logistic regression was used for the binary frailty outcome, and linear regression for the continuous frailty index. Model 1 was unadjusted. Model 2 adjusted for age, gender, education, marital status, residence, and living arrangement. Model 3 further adjusted for smoking, drinking, exercise, social participation, kitchen’s ventilation status, and method for cooking, mold exposure, residential distance from the main road, and ambient concentrations of CO, NO₂, O₃, PM_2.5_, PM_10_, and SO_2_. Additionally, based on Model 3, both linear and logistic regression models were used to analyze seasonal variations in the association between IVF and frailty. Subgroup and interaction analyses were conducted to explore potential effect modification by various population characteristics. To mitigate multiple comparison concerns and control the false positive rate, false discovery rate correction was applied to all *p*-values from subgroup analyses (29). The same analytical procedures were applied to the external validation dataset from Chongqing. All statistical analyses were performed using R version 4.3.0 and SPSS version 25.0. A two-sided *p*-value < 0.05 was considered statistically significant.

## 3 Results

### 3.1 Basic characteristics description

Among the 5,511 participants from the CLHLS survey, 1,568 (28.45%) were identified as frail. The sample comprised 2,595 males (47.07%) and 2,917 females (52.93%). A total of 2,405 (43.64%) participants had no formal education, 2,419 (43.89%) resided in rural areas, and 4,630 (84.01%) lived with family. The numbers of older adults who smoke, drink, exercise, and good social participation were 937 (17.00%), 912 (16.55%), 2,034 (36.91%), and 911 (16.53%), respectively. Regarding air pollution exposure, 3,372 (61.19%) used non-clear energy for cooking, 472 (8.56%) had poor kitchen ventilation, 726 (13.17%) reported mold exposure, and 2,270 (41.19%) lived within 200 meters of a major road. Frailty status differed significantly across categories of age, gender, education, marital status, residence, living arrangement, smoking, drinking, exercise, and social participation (*p* < 0.05). Significant variations in frailty were also observed across different concentrations of NO₂, O₃, PM₂.₅, and PM₁₀ (*p* < 0.05) (Table 1).

**Table 1:** Basic characteristics of the study participants at baseline by frailty (*n* = 5,511).

Variables	Total (*n* = 5,511)	Normal (*n* = 3,943)	Frailty (*n* = 1,568)	Statistic	*P*
Age, M (Q₁, Q₃)	82.00 (73.00, 91.00)	78.00 (71.00, 86.00)	93.00 (86.00, 100.00)	*Z* = −37.19	**<0.001**
Gender, *n*(%)				*χ*^2^ = 90.51	**<0.001**
Female	2,917 (52.93)	1,928 (48.90)	989 (63.07)		
Male	2,594 (47.07)	2,015 (51.10)	579 (36.93)		
Education, *n*(%)				*χ*^2^ = 378.76	**<0.001**
0 years	2,405 (43.64)	1,401 (35.53)	1,004 (64.03)		
≤6 years	2,001 (36.31)	1,603 (40.65)	398 (25.38)		
>6 years	1,105 (20.05)	939 (23.81)	166 (10.59)		
Marital status, *n*(%)				*χ*^2^ = 491.10	**<0.001**
Other	2,808 (50.95)	1,638 (41.54)	1,170 (74.62)		
Married	2,703 (49.05)	2,305 (58.46)	398 (25.38)		
Residence, *n*(%)				*χ*^2^ = 4.00	**0.045**
Rural	2,419 (43.89)	1,764 (44.74)	655 (41.77)		
City	3,092 (56.11)	2,179 (55.26)	913 (58.23)		
Living arrangement, *n*(%)				*χ*^2^ = 36.99	**<0.001**
Living with family	4,630 (84.01)	3,240 (82.17)	1,390 (88.65)		
Living alone	871 (15.81)	697 (17.68)	174 (11.10)		
Living in institution	10 (0.18)	6 (0.15)	4 (0.26)		
Smoking, *n*(%)				*χ*^2^ = 43.09	**<0.001**
No	4,574 (83.00)	3,190 (80.90)	1,384 (88.27)		
Yes	937 (17.00)	753 (19.10)	184 (11.73)		
Drinking, *n*(%)				*χ*^2^ = 67.79	**<0.001**
No	4,599 (83.45)	3,188 (80.85)	1,411 (89.99)		
Yes	912 (16.55)	755 (19.15)	157 (10.01)		
Exercise, *n*(%)				*χ*^2^ = 297.37	**<0.001**
No	3,477 (63.09)	2,209 (56.02)	1,268 (80.87)		
Yes	2,034 (36.91)	1,734 (43.98)	300 (19.13)		
Social participant, *n*(%)				*χ*^2^ = 198.29	**<0.001**
Poor	4,600 (83.47)	3,116 (79.03)	1,484 (94.64)		
Good	911 (16.53)	827 (20.97)	84 (5.36)		
Method for cooking, *n*(%)				*χ*^2^ = 3.47	0.062
Non-clean energy	3,372 (61.19)	2,443 (61.96)	929 (59.25)		
Clean energy	2,139 (38.81)	1,500 (38.04)	639 (40.75)		
Kitchen’s ventilation status, *n*(%)				*χ*^2^ = 0.12	0.725
No	472 (8.56)	341 (8.65)	131 (8.35)		
Yes	5,039 (91.44)	3,602 (91.35)	1,437 (91.65)		
Mold exposure, *n*(%)				*χ*^2^ = 0.56	0.456
No	4,785 (86.83)	3,432 (87.04)	1,353 (86.29)		
Yes	726 (13.17)	511 (12.96)	215 (13.71)		
Distance of residence from the main road, *n*(%)				*χ*^2^ = 2.28	0.131
<200 meters	2,270 (41.19)	1,649 (41.82)	621 (39.61)		
≥200 meters	3,241 (58.81)	2,294 (58.18)	947 (60.40)		
CO, M (Q₁, Q₃)	0.87 (0.81, 0.93)	0.87 (0.81, 0.93)	0.865 (0.81, 0.93)	*Z* = −1.72	0.085
NO_2_, M (Q₁, Q₃)	26.40 (18.95, 34.27)	25.27 (18.95, 34.27)	29.91 (19.51, 34.36)	*Z* = −4.14	**<0.001**
O_3_, M (Q₁, Q₃)	98.00 (89.08, 108.57)	98.00 (89.08, 108.57)	102.28 (91.27, 108.57)	*Z* = −3.27	**0.001**
PM_2.5_, M (Q₁, Q₃)	38.12 (30.07, 46.22)	34.89 (29.90, 46.22)	43.85 (30.07, 46.22)	*Z* = −2.77	**0.006**
PM_10_, M (Q₁, Q₃)	66.78 (48.90, 83.66)	64.36 (48.90, 78.85)	72.99 (48.90, 86.35)	*Z* = −3.95	**<0.001**
SO_2_, M (Q₁, Q₃)	12.32 (10.87, 12.52)	12.32 (10.87, 12.52)	12.32 (10.67, 14.40)	*Z* = −0.81	0.416

Z, Mann–Whitney test, *χ*^2^, Chi-square test.

M, Median, Q₁, 1st Quartile, Q₃, 3st Quartile.

Bold value indicates *p* < 0.05.

Based on ventilation frequency, participants were categorized into low (*n* = 467), intermediate (*n* = 1,701), and high (*n* = 3,343) IVF groups. Significant differences in IVF levels were observed across age, education, marital status, residence, living arrangement, exercise, social participation, cooking method, kitchen’s ventilation status, and mold exposure (*p* < 0.05). Moreover, all air quality indicators (CO, NO_2_, O_2_, PM_2.5_, PM_10_, and SO_2_) were significantly associated with ventilation frequency (*p* < 0.001) (Supplementary Table 3). We further explored the basic characteristics of the association between different frequencies of IVF in each season and frailty. Among them, the number of older adults with a ventilation frequency >5 times/week in spring, summer, autumn, and winter was 3,237 (58.74%), 4,308 (78.17%), 3,271 (59.35%), and 2,119 (38.45%), respectively. Except for spring, different ventilation frequencies in autumn, summer, and winter were significantly associated with frailty (*p* < 0.05). The detailed results are presented in Table 2.

**Table 2:** Bivariate association between Seasonal ventilation frequency and frailty.

Variables	Total (*n* = 5,511)	Normal (*n* = 3,943)	Frailty (*n* = 1,568)	Statistic	*P*
Spring ventilation frequency, *n*(%)				*χ*^2^ = 4.934	0.085
0 time/week	250 (4.54)	164 (4.16)	86 (5.49)		
1–5 times/week	2,024 (36.73)	1,444 (36.62)	580 (36.99)		
>5 times/week	3,237 (58.74)	2,335 (59.22)	902 (57.53)		
Summer ventilation frequency, *n*(%)				*χ*^2^ = 7.289	**0.026**
0 time/week	153 (2.78)	98 (2.49)	55 (3.51)		
1–5 times/week	1,050 (19.05)	731 (18.54)	319 (20.34)		
>5 times/week	4,308 (78.17)	3,114 (78.98)	1,194 (76.15)		
Autumn ventilation frequency, *n*(%)				*χ*^2^ = 7.540	**0.023**
0 time/week	214 (3.88)	140 (3.55)	74 (4.72)		
1–5 times/week	2,026 (36.76)	1,425 (36.14)	601 (38.33)		
>5 times/week	3,271 (59.35)	2,378 (60.31)	893 (56.95)		
Winter ventilation frequency, *n*(%)				*χ*^2^ = 11.761	**0.003**
0 time/week	1,154 (20.94)	783 (19.86)	371 (23.66)		
1–5 times/week	2,238 (40.61)	1,644 (41.69)	594 (37.88)		
>5 times/week	2,119 (38.45)	1,516 (38.45)	603 (38.46)		
Overall ventilation index, *n*(%)				*χ*^2^ = 12.056	**0.002**
0–3 (low)	467 (8.47)	303 (7.69)	164 (10.46)		
4–5 (intermediate)	1,701 (30.87)	1,213 (30.76)	488 (31.12)		
6–8 (high)	3,343 (60.66)	2,427 (61.55)	916 (58.42)		

*χ*^2^, Chi-square test.

Bold value indicates *p* < 0.05.

To ensure that our findings were not attributable to chance or specific to the CLHLS dataset, an external validation was performed using survey data from 718 older adults recruited from Chongqing Geriatric Hospital between June and July 2025. Sample characteristics are detailed in Supplementary Table 4. Among these participants, 176 (24.51%) were frail. The sample comprised 335 males (46.66%) and 383 females (53.34%), with the majority aged 82 years or older. Significant differences between frail and non-frail groups were observed in age, gender, education, marital status, drinking, exercise, and social participation (*p* < 0.05).

### 3.2 Association between indoor ventilation frequency and frailty

Logistic regression analysis indicated that IVF was negatively correlated with the binary frailty risk (Table 3). In Model 1, without adjusting for any variables, compared with the low frequency group, the prevalence of frailty in the intermediate and high IVF groups was significantly reduced (Intermediate IVF: OR = 0.743, 95% CI: 0.598 ~ 0.924; High IVF: OR = 0.697, 95% CI: 0.568 ~ 0.856). In Model 2, which further considered age, gender, education, marital status, residence, and living arrangement, the prevalence of frailty among older adults with intermediate and high IVF decreased by 27.6% (OR = 0.724, 95% CI: 0.565 ~ 0.927) and 25.3% (OR = 0.747, 95% CI: 0.590 ~ 0.946), respectively. As more covariates were included in the models, this negative association slightly attenuated but remained statistically significant. The results of Model 3, which fully adjusted for all confounding factors, showed that the OR values for intermediate and high IVF were 0.722 (95% CI: 0.559 ~ 0.933) and 0.824 (95% CI: 0.643 ~ 0.995), respectively. Additionally, the linear regression model further confirmed that a higher IVF was significantly associated with a lower frailty index, with 20 covariates included as confounding factors in the model. For the intermediate ventilation frequency, compared with the low frequency group, the frailty index of individuals in the intermediate IVF group was significantly lower (Beta coefficients = −0.02; 95% CI: −0.03 ~ −0.01; *p* < 0.001), and the high IVF group also showed a significant negative association (Beta coefficients = −0.01; 95% CI: −0.03 ~ −0.01; *p* < 0.01). The detailed results are presented in Table 4.

**Table 3:** The association between indoor ventilation frequency and frailty (categorical).

	Low frequency	Intermediate frequency	High frequency
	OR (95% CI)		
Model 1	Ref.	0.743 (0.598, 0.924)**	0.697 (0.568, 0.856)***
Model 2	Ref.	0.724 (0.565, 0.927)*	0.747 (0.590, 0.946)*
Model 3	Ref.	0.722 (0.559, 0.933)*	0.824 (0.643, 0.995)*

OR is odds ratio; CI is confidence interval; *p < 0.05, **p < 0.01, ***p < 0.001.

Model 1: Crude model.

Model 2: Adjusted for age, gender, education, marital status, residence, and living arrangement.

Model 3: Adjusted for model 2 + smoking, drinking, exercise, social participation, methods for cooking, kitchen’s ventilation status, mold exposure, distance of residence from the main road, concentrations of CO, NO_2_, O_3_, PM_2.5_, PM_10_, and SO_2_.

**Table 4:** The association between indoor ventilation frequency and frailty (continuous).

	Low frequency	Intermediate frequency	High frequency
	Beta coefficients (95% CI)		
Model 1	Ref.	−0.03 (−0.04, −0.01)***	−0.03 (−0.05, −0.02)***
Model 2	Ref.	−0.02 (−0.04, −0.01)***	−0.02 (−0.03, −0.01)***
Mode 3	Ref.	−0.02 (−0.03, −0.01)***	−0.01 (−0.03, −0.01)**

CI is confidence interval; **p < 0.01, ***p < 0.001.

Model 1: Crude model.

Model 2: Adjusted for age, gender, education, marital status, residence, and living arrangement.

Model 3: Adjusted for model 2 + smoking, drinking, exercise, social participation, methods for cooking, kitchen’s ventilation status, mold exposure, distance of residence from the main road, concentrations of CO, NO_2_, O_3_, PM_2.5_, PM_10_, and SO_2_.

To validate the robustness of the above findings, we conducted an external validation in an independent sample from Chongqing. The results replicated the core conclusions (Supplementary Tables 5, 6). Logistic regression showed that compared with low IVF, both intermediate (OR = 0.975, 95% CI: 0.485 ~ 0.993) and high (OR = 0.952, 95% CI: 0.491 ~ 0.997) IVF were associated with a lower risk of frailty. Linear regression analysis also indicated that a higher IVF was significantly associated with a lower continuous frailty index.

### 3.3 Association between seasonal indoor ventilation frequency and frailty

To further comprehensively evaluate the seasonal variation, we used logistic and linear regression models for analysis. The seasonal stratified models in this study were built based on Model 3, which has indirectly adjusted for the potential confounding effect of seasonal differences in pollutant levels by incorporating regional pollutant concentrations (CO, NO₂, O₃, PM_2.5_, PM_10_, and SO_2_). When frailty was considered as a binary variable, the logistic regression results showed that a higher ventilation frequency had a protective effect against frailty in specific seasons. Compared with low IVF, intermediate IVF in winter (OR = 0.837, 95% CI: 0.695 ~ 0.887) and high IVF in summer (OR = 0.809, 95% CI: 0.545 ~ 0.953), autumn (OR = 0.887, 95% CI: 0.628 ~ 0.922), and winter (OR = 0.835, 95% CI: 0.715 ~ 0.975) were all associated with a reduced risk of frailty (*p* < 0.05). This suggests that maintaining winter ventilation, especially at an intermediate frequency, may be most closely related to a reduced risk of frailty (Supplementary Table 7).

The linear regression model revealed more subtle associations between seasonal ventilation frequency and continuous frailty. Compared with low IVF, intermediate IVF in spring (Beta coefficients = −0.03, 95% CI: −0.03 ~ −0.01), autumn (Beta coefficients = −0.01, 95% CI: −0.03 ~ −0.00), and winter (Beta coefficients = −0.01, 95% CI: −0.02 ~ −0.01) were significantly associated with a lower frailty index. However, the beneficial association of high IVF was only statistically significant in autumn (Beta coefficients = −0.01, 95% CI: −0.03 ~ −0.01). This indicates that for the continuous change in the degree of frailty, intermediate IVF shows benefits in most seasons, but the effect size is generally small (Supplementary Table 8).

### 3.4 Subgroup analyses and interaction analyses

**Figure 3:**
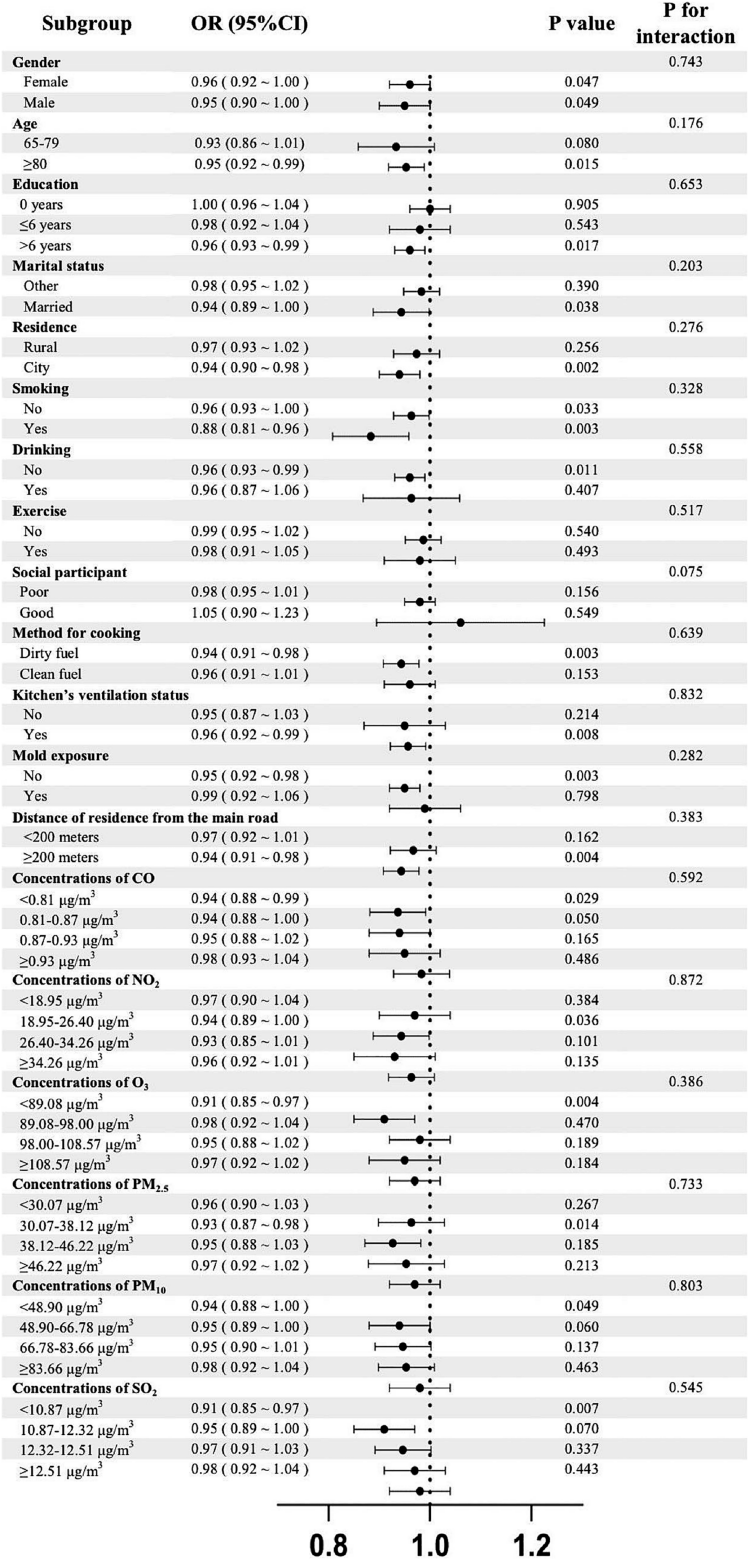
Results of subgroup analyses about the association of indoor ventilation frequency and frailty.

The results of subgroup analysis showed that the association between IVF and frailty appeared more pronounced among older adults of different genders, those over 80 years old, with more than 6 years of education, married, living in urban areas, smoking or non-current smoking, non-current drinking, using non-clean energy, having kitchen’s ventilation equipment, without mold exposure, and living at a distance of ≥ 200 meters from the main road, with CO < 0.81 μg/m^3^, NO_2_ = 18.95 ~ 26.40 μg/m^3^, O_3_ < 89.08 μg/m^3^, PM_2.5_ = 30.07 ~ 38.12 μg/m^3^, PM_10_ < 48.90 μg/m^3^, and SO_2_ < 10.87 μg/m^3^. Interaction analysis did not identify significant modifiers. All results are presented in Figure 3.

## 4 Discussion

Frailty represents a significant global health burden due to its strong association with adverse outcomes in older adults, including falls, disability, and diminished quality of life (8–10). Extensive prior research has indicated that air pollutants may be linked to a variety of diseases. Due to their fine particle size, PM_2.5_ can penetrate deeply into the alveolar region (30), triggering multiple deleterious pathways related to inflammation and cellular damage through mechanisms such as oxidative stress and inflammatory responses, ultimately leading to pulmonary injury and exacerbating respiratory conditions like chronic obstructive pulmonary disease, asthma, and pulmonary fibrosis (31, 32). Previous studies have also demonstrated that PM_2.5_ increases susceptibility to bacterial and viral infections by altering levels of pro-inflammatory cytokines (e.g., IL-1β, IL-6, and IFN-β), thereby elevating the risk of frailty (33, 34). Furthermore, associations between other air pollutants—such as CO, NO₂, and SO₂—and various diseases have been corroborated in other studies (35, 36). These pollutants tend to accumulate in poorly ventilated indoor environments, substantially increasing the risk of cardiovascular and respiratory diseases among older adults (37). Given that individuals spend over 90% of their time in enclosed spaces, maintaining good indoor air quality and reducing potential pollutants may play a positive role in preventing and mitigating adverse health outcomes among older adults (17, 28). Using data from the CLHLS, this study is the first to reveal a significant association between IVF and frailty in older adults, with external validation data from Chongqing further affirming the robustness of the findings. Against the backdrop of a progressively aging global population, this research provides new evidence for evidence-based medicine and holds important implications for promoting healthy and active aging.

This study is the first to identify a association between IVF and frailty. Specifically, ventilating more than five times per week was linked to a statistically significant reduction in frailty risk among older adults. However, due to the cross-sectional design of the study, the temporal sequence remains uncertain, allowing for two plausible interpretations. On one hand, higher IVF may reduce the accumulation of indoor allergens such as bacteria, viruses, and mold, thereby improving indoor air quality, alleviating allergy and asthma symptoms, and ultimately lowering frailty risk (38, 39). On the other hand, reverse causality may also be at play: older adults in poorer health may be more susceptible to environmental pollutants (e.g., CO₂, VOCs) and thus reduce ventilation frequency due to factors such as chill sensitivity or being largely homebound (40, 41). Linear regression results indicated that each one-unit increase in IVF was associated with a 2% reduction in the risk of frailty among older adults (*β* = −0.02). Although the effect size is modest, we note that effect sizes of *β* < 0.10 are commonly observed in sociological and psychological research and still hold clinical relevance. A prior study involving 2,271 older adults found that living alone (*β* = 0.011) and personal social networks (*β* = 0.005) exerted significant mediating effects on the relationship between physical frailty and basic activities of daily living limitations (42). Similarly, a cohort study based on the Korean Frailty and Aging Cohort Study (KFACS) reported that each additional fall experience in early old age was associated with an approximate 4.1% increase in frailty risk in later life (*β* = 0.041) (43). Therefore, we attribute the observed modest effect to the multifactorial etiology of frailty—specifically, its development typically results from the interplay of multiple risk factors. Moreover, from a public health prevention and intervention perspective, IVF-based interventions are highly feasible, low-cost, and free of adverse effects, rendering them practically valuable. Secondly, our study population represents a nationally representative sample of older adults. According to the Seventh National Population Census of China in 2020, the population aged 60 and above has reached 264 million (44). Applying the concept of population attributable risk, even a risk factor with a small effect size may exert substantial population-level impact if it affects a large number of individuals. Finally, the potential cumulative effect of minimal risks must be considered. Long-term exposure to slight risk factors, or the combined influence of multiple such factors, may lead to significant health consequences over time. Whether such effects can accumulate through sustained intervention warrants validation in future longitudinal cohort studies.

The effect of IVF on frailty exhibited significant seasonal variation, with the strongest associations observed in autumn and winter, and weaker effects in spring and summer. This pattern may be attributable to the naturally better ventilation conditions in warmer seasons. During spring and summer, outdoor air quality in most regions of China (excluding heavily polluted industrial areas) is generally favorable, with comfortable temperatures and adequate air movement, meaning even modest ventilation can maintain acceptable indoor air quality. Consequently, differences in ventilation frequency may yield relatively minor disparities in indoor air quality improvement during these seasons. In contrast, autumn and winter are peak seasons for respiratory infections such as influenza and bronchitis (45). Poorly ventilated enclosed spaces provide ideal conditions for pathogen transmission, and consistent ventilation helps dilute pathogen concentration and prevent infections. For older adults, a severe respiratory infection can be a critical event leading to frailty progression (46). Additionally, the association between IVF and frailty was particularly pronounced in winter. Indoor pollutants such as PM_2.5_ and CO often reach higher concentrations in winter due to reduced window opening in cold weather and the use of heating appliances. Thus, maintaining ventilation during winter may contribute to better indoor air quality and lower frailty risk. From a physiological standpoint, cold exposure can induce peripheral vasoconstriction, elevated blood pressure, and increased heart rate, thereby raising cardiac afterload and myocardial oxygen demand, which places additional strain on older adults and may precipitate frailty and elevate cardiovascular risk (47, 48). In this context, prolonged window closure due to cold sensitivity may lead to accumulation of indoor CO₂. Elevated CO₂ levels have been linked to reduced cerebral blood flow, changes in intracranial pressure, and declines in cognitive function, along with increased drowsiness and fatigue in older adults (49, 50). These effects can reduce daily physical activity, which may in turn contribute to frailty onset, creating a vicious cycle (51). Therefore, maintaining adequate indoor ventilation during winter is crucial for frailty prevention.

The association between IVF and frailty among Chinese older adults was heterogeneous across different population subgroups. Compared with males, IVF had a more significant effect on the probability of frailty in older females. Due to aging, especially after menopause, the hormone levels of older women will decrease, which will further lead to lower blood pressure and reduced thermoregulation (52). In addition, data show that older women always have a lower level of physical activity and suffer from a higher proportion of chronic diseases, such as cardiovascular diseases and respiratory diseases (53, 54). Weaker thermoregulation and the occurrence of more chronic diseases may make their bodies more vulnerable to adverse environmental factors that lead to physical discomfort, increasing the prevalence of frailty. Older age is also a risk factor for frailty. With the increase in age, the decline of organ function among older adults is increasingly serious (55). An environment with poor IVF can lead to lower oxygen levels in the air and higher carbon dioxide concentrations, which are more likely to cause breathing difficulties and hypoxia in older people whose lung function is already declining. At the same time, older adults with declining cardiovascular function also more difficult to adapt to temperature changes. The poor IVF environment may lead to an increase in indoor temperature, thereby increasing the cardiovascular burden and inducing cardiovascular disease. Combined with the effects of poorer immune system function, they are often sicker and more difficult to recover from once infected. Previous studies have also confirmed that smoking and drinking significantly increase the prevalence of frailty among older adults and may even increase the risk of death (56–58). However, the effect of increasing IVF to alleviate frailty in old age is relatively limited and has little impact on people who enjoy smoking and drinking on a daily basis. Therefore, older adults who do not smoke and drink tend to be more likely to maintain a healthier lifestyle through increased IVF with a lower prevalence of chronic disease and a reduced incidence of frailty than those who do. Because of their better physical foundation, these healthy older adults may be more sensitive to subtle changes in environmental quality and more likely to detect increased concentrations of harmful substances in poorly ventilated environments, which may lead to a debilitating outcome.

The protective association between IVF and frailty was more pronounced and statistically significant among older adults who used non-clear energy (e.g., coal, wood) for cooking, whereas it was attenuated and non-significant among clean energy users. This pattern is mechanistically plausible. The combustion of non-clear energy is a potent source of various hazardous air pollutants, including carbon monoxide, sulfur dioxide, and PM_2.5_ (59). In inadequately ventilated indoor environments, these pollutants accumulate, substantially elevating the risk of cardiovascular and respiratory diseases (37), which may, in turn, accelerate the progression of frailty. This offers a potential explanation for the modification effect observed. Similarly, the association was more evident among those with kitchen ventilation, without a history of mold exposure, and residing ≥200 m from a major road. During cooking, concentrated pollutants (e.g., PM_2.5_, NO₂, and VOCs) are generated. In the absence of localized exhaust (e.g., a range hood), general room ventilation through windows may be insufficient to effectively and continuously remove this point-source pollution, potentially offsetting the health benefits of ventilation. This may clarify why the association was primarily detectable in households with kitchen ventilation. The mechanism for mold exposure is analogous but distinct. Molds release spores and mycotoxins (e.g., containing β-glucans) that are potent immunostimulants and inflammatory triggers (60, 61). The resultant health threat evolves from general air pollution to a persistent biological inflammatory assault, linked to respiratory and immune disorders (62, 63), which can chronically promote muscle loss and frailty. In such scenarios, simply diluting contaminants via window ventilation is likely inadequate to counteract the intense and ongoing pathophysiological process driven by the mold itself, potentially explaining the null association in the mold-exposed subgroup. Regarding proximity to major roads, for individuals living ≥200 m away, outdoor air is relatively cleaner. Opening windows in this context effectively removes indoor pollutants while introducing fresher air, making the protective effect of IVF more discernible. Conversely, for those living near heavy traffic, frequent ventilation might introduce significant outdoor pollutants (e.g., vehicle exhaust), which could counterbalance the benefits of removing indoor pollutants, thereby obscuring a clear association with frailty. Crucially, however, the interpretation of these subgroup findings requires caution. First, the interaction terms for all the aforementioned subgroup variables were statistically non-significant, substantially weakening the evidence for true effect modification. Second, some strata (e.g., current smokers, clean energy users) had limited sample sizes, which can lead to unstable estimates and inflated effect sizes. Therefore, these observed patterns should be regarded strictly as exploratory and hypothesis-generating. They highlight potential susceptible populations and suggest mechanistic pathways that warrant verification in future, larger longitudinal studies with sufficient statistical power.

## 5 Conclusion

This study is the first to systematically demonstrate a significant association between IVF and frailty among Chinese older adults, and this finding was validated in an independent external data. The study further analyzed the seasonal differences in this association, with more significant results in autumn and winter. Additionally, subgroup analysis revealed differences in the association between IVF and frailty in terms of gender, age, and lifestyle, suggesting that when formulating treatments or policies to improve the frailty status of older adults, more attention should be paid to groups such as female, those over 80 years old, those without mold exposure experience, and those living at a distance of ≥200 meters from the main road. This study provides strong evidence for the protective effect of air circulation on the frailty status of older adults. Moreover, as a potential intervention measure with low cost, easy implementation, and safety, IVF has certain reference value for clinical treatment and policy-making, which has positive practical significance for promoting healthy aging.

## 6 Limitation

This study has several limitations. Firstly, the cross-sectional design prevents the study from directly proving a causal association between IVF and frailty and from capturing the temporal relationship between frailty and indoor ventilation. Second, the assessment of IVF relied on self-reported weekly window-opening frequency, which did not account for duration, time points, or the usage of supplementary ventilation equipment. Such undifferentiated misclassification is likely to result in an underestimate of the effect size. Finally, although we adjusted for a wide range of covariates, several other potential factors that might influence the association between IVF and frailty, such as ambient temperature, building orientation, floor level, and number of windows, were not included in the model due to limitations of the public database. This may have affected the accuracy of the results to some extent.

## Data Availability

Publicly available datasets were analyzed in this study. This data can be found at: (https://opendata.pku.edu.cn).

## Footnotes

1
https://weijing-rs.github.io/product.html
