# Supplementary material for: Association between indoor ventilation frequency and frailty among Chinese older adults
Source: Front Public Health. 2025 Dec 1;13:1670577. doi: 10.3389/fpubh.2025.1670577 (PMC12704094; doi:10.3389/fpubh.2025.1670577)
Supplement: Supplementary file 1 [file Supplementary_file_1.docx]

Supplementary Material

Supplementary Figure 1. Lowess smooth plot of the association between continuous indoor ventilation frequency score and the frailty.


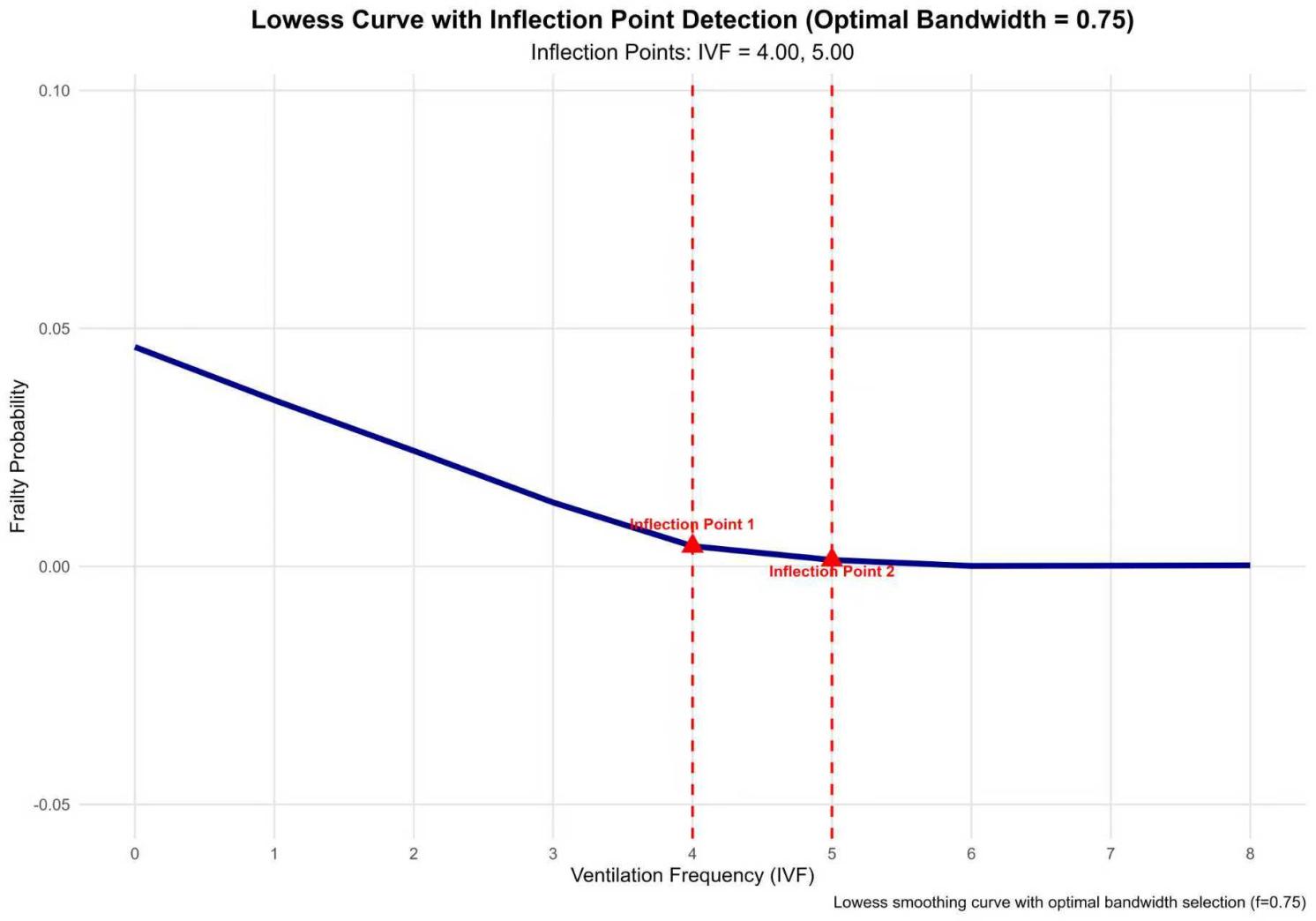


Supplementary Table 1. Questionnaire items and classification criteria of covariates

| **Variables** | **Classification** |
| --- | --- |
| Age | Continuous Variable |
| Gender | 0 = Female, 1 = Male |
| Education | 0 = 0 years, 1 = ≤6 years, 2 = >6 years |
| Marital status | 0 = Other, 1 = Married |
| Residence | 0 = Rural, 1 = City |
| Living arrangement | 0 = Living with family, 1 = Living alone, 2 = Living in institution |
| Smoking | 0 = No, 1 = Yes |
| Drinking | 0 = No, 1 = Yes |
| Exercise | 0 = No, 1 = Yes |
| Social participant | 0 = Poor, 1 = Good |
| Method for cooking | 0 = Nonclean energy, 1 = Clean energy |
| Kitchen’s ventilation status | 0 = No, 1 = Yes |
| Mold exposure | 0 = No, 1 = Yes |
| Distance of residence from the main road | 0 = <200 meters, 1 = ≥200 meters |
| Concentrations of CO, n(%) | 0 = <0.81 μg/m^3^, 1 = 0.81-0.87 μg/m^3^, 2 = 0.87-0.93 μg/m^3^, 3 = ≥0.93 μg/m^3^ |
| Concentrations of NO_2_, n(%) | 0 = <18.95 μg/m^3^, 1 = 18.95-26.40 μg/m^3^, 2 = 26.40-34.26 μg/m^3^, 3 = ≥34.26 μg/m^3^ |
| Concentrations of O_3_, n(%) | 0 = <89.08 μg/m^3^, 1 = 89.08-98.00 μg/m^3^, 2 = 98.00-108.57 μg/m^3^, 3 = ≥108.57 μg/m^3^ |
| Concentrations of PM_2.5_, n(%) | 0 = <30.07 μg/m^3^, 1 = 30.07-38.12 μg/m^3^, 2 = 38.12-46.22 μg/m^3^, 3 = ≥46.22 μg/m^3^ |
| Concentrations of PM_10_, n(%) | 0 = <48.90 μg/m^3^, 1 = 48.90-66.78 μg/m^3^, 2 = 66.78-83.66 μg/m^3^, 3 = ≥83.66 μg/m^3^ |
| Concentrations of SO_2_, n(%) | 0 = <10.87 μg/m^3^, 1 = 10.87-12.32 μg/m^3^, 2 = 12.32-12.51 μg/m^3^, 3 = ≥12.51 μg/m^3^ |

Supplementary Table 2. Questions and coding of variables about the Frailty Index scale

| **List** | **Definition according to baseline self-report or physical measurements** | **Coding of variables** | | **15874** |
| --- | --- | --- | --- | --- |
| 1 | IADLs: Unable to visit neighbors by oneself--e7 | yes=1 | no=0 | 15,725 |
| 2 | IADLs: Unable to shop by oneself if necessary--e8 | yes=1 | no=0 | 15,720 |
| 3 | IADLs: Unable to cook meals by oneself if necessary--e9 | yes=1 | no=0 | 15,714 |
| 4 | IADLs: Unable to wash clothing by oneself--e10 | yes=1 | no=0 | 15,708 |
| 5 | IADLs: Unable to walk continuously for 1 kilometer--e11 | yes=1 | no=0 | 15,706 |
| 6 | IADLs: Unable to lift a weight of 5 kg (such as a heavy bag of groceries)--e12 | yes=1 | no=0 | 15,691 |
| 7 | IADLs: Unable to continuously crouch and stand up three times--e13 | yes=1 | no=0 | 15,646 |
| 8 | IADLs: Unable to use public transportation--e14 | yes=1 | no=0 | 15,619 |
| 9 | Functional limitations: Unable to put hand behind neck--g81 | yes=1 | no=0 | 15,484 |
| 10 | Functional limitations: Unable to put hand behind lower back--g82 | yes=1 | no=0 | 15,439 |
| 11 | Functional limitations: Unable to raise arm upright--g83 | yes=1 | no=0 | 14285 |
| 12 | Functional limitations: Unable to stand up from sitting in a chair--g9 | yes=1 | no=0 | 14,216 |
| 13 | Functional limitations: Unable to pick up a book from the floor--g11 | yes=1 | no=0 | 14,129 |
| 14 | ADLs: Needs assistance bathing--e1 | yes=1 | no=0 | 14,083 |
| 15 | ADLs: Needs assistance dressing--e2 | yes=1 | no=0 | 13,984 |
| 16 | ADLs: Needs assistance toileting--e3 | yes=1 | no=0 | 13,957 |
| 17 | ADLs: Needs assistance going in indoors--e4 | yes=1 | no=0 | 13,868 |
| 18 | ADLs: Needs assistance eating--e5 | yes=1 | no=0 | 13,835 |
| 19 | ADLs: Needs Incontinence--e6 | yes=1 | no=0 | 13,584 |
| 20 | Cognitively impaired (based on the Mini-Mental State Examination) | yes=1 | no=0 | 9,930 |
| 21 | Poor self-rated health--b12 | yes=1 | no=0 | 9,567 |
| 22 | Health worsened in the past year--b121 | yes=1 | no=0 | 9,526 |
| 23 | Poor interview-rated health--h3 | yes=1 | no=0 | 9,468 |
| 24 | Hearing loss--h1 | yes=1 | no=0 | 9,450 |
| 25 | Vision loss--g1 | yes=1 | no=0 | 9,400 |
| 26 | Abnormal heart rhythm--g20 | yes=1 | no=0 | 9,400 |
| 27 | Symptom of psychological distress (based on loneliness, usefulness, and fearfulness)--b34/b36/b38 | yes=1 | no=0 | 8,963 |
| 28 | Number of serious illnesses in the past 2 years**^ａ^--g131** | yes=1/2 | no=0 | 8,641 |
| 29 | Suffering from hypertension, systolic blood pressure measured to be ≥140 mm Hg, or diastolic blood pressure measured to be ≥90mmHg  --g15a1/g511/g512/g521/g522 | yes=1 | no=0 | 7,410 |
| 30 | Suffering from diabetes--g15b1 | yes=1 | no=0 | 7,072 |
| 31 | Suffering from tuberculosis--g15f1 | yes=1 | no=0 | 6,974 |
| 32 | Suffering from heart disease--g15c1 | yes=1 | no=0 | 6,925 |
| 33 | Suffering from stroke/cerebrovascular disease--g15d1 | yes=1 | no=0 | 6,759 |
| 34 | Suffering from bronchitis, emphysema, asthma, or pneumonia--g15e1 | yes=1 | no=0 | 6,735 |
| 35 | Suffering from cancer--g15i1 | yes=1 | no=0 | 6,402 |
| 36 | Suffering from arthritis--g15n1 | yes=1 | no=0 | 6,377 |
| 37 | Suffering from bedsores--g15m1 | yes=1 | no=0 | 6,375 |
| 38 | Suffering from gastric or duodenal ulcers--g15k1 | yes=1 | no=0 | 6,359 |
| 39 | Suffering from Parkinson's disease--g15l1 | yes=1 | no=0 | 6,350 |

**Note:** IADLs= instrumental activities of daily living; ADLs= activities of daily

^a^ Persons reporting two or more illnesses are assigned a value of 2.

**Supplementary Table 3**. Basic characteristics among different indoor ventilation frequency groups (n=5,511).

| **Variables** | **Low frequency**  **(n = 467)** | **Intermediate**  **Frequency**  **(n = 1,701)** | **High frequency**  **(n = 3,343)** | **Statistic** | ***P*** |
| --- | --- | --- | --- | --- | --- |
|  |  |  |  |  |  |
| Age, M (Q₁, Q₃) | 85.00 (75.00,93.00) | 83.00 (74.00,92.00) | 81.00 (73.00,91.00) | χ²=19.23# | **<.001** |
| Gender, n(%) |  |  |  | χ²=5.46 | 0.065 |
| Female | 267 (57.17) | 916 (53.85) | 1,734 (51.87) |  |  |
| Male | 200 (42.83) | 785 (46.15) | 1,609 (48.13) |  |  |
| Education, n(%) |  |  |  | χ²=146.33 | **<.001** |
| 0 years | 284 (60.81) | 830 (48.80) | 1,291 (38.62) |  |  |
| ≤6 years | 134 (28.69) | 625 (36.74) | 1,242 (37.15) |  |  |
| >6 years | 49 (10.49) | 246 (14.46) | 810 (24.23) |  |  |
| Marital status, n(%) |  |  |  | χ²=12.43 | **0.002** |
| Other | 266 (56.96) | 895 (52.62) | 1,647 (49.27) |  |  |
| Married | 201 (43.04) | 806 (47.38) | 1,696 (50.73) |  |  |
| Residence, n(%) |  |  |  | χ²=118.84 | **<.001** |
| Rural | 269 (57.60) | 874 (51.38) | 1,276 (38.17) |  |  |
| City | 198 (42.40) | 827 (48.62) | 2,067 (61.83) |  |  |
| Living arrangement, n(%) |  |  |  | - | **<.001*** |
| Living with family | 358 (76.66) | 1,416 (83.25) | 2,856 (85.43) |  |  |
| Living alone | 109 (23.34) | 282 (16.58) | 480 (14.36) |  |  |
| Living in institution | 0 (0.00) | 3 (0.18) | 7 (0.21) |  |  |
| Smoking, n(%) |  |  |  | χ²=1.92 | 0.382 |
| No | 395 (84.58) | 1,422 (83.60) | 2,757 (82.47) |  |  |
| Yes | 72 (15.42) | 279 (16.40) | 586 (17.53) |  |  |
| Drinking, n(%) |  |  |  | χ²=2.89 | 0.235 |
| No | 396 (84.80) | 1,436 (84.42) | 2,767 (82.77) |  |  |
| Yes | 71 (15.20) | 265 (15.58) | 576 (17.23) |  |  |
| Exercise, n(%) |  |  |  | χ²=114.42 | **<.001** |
| No | 349 (74.73) | 1,204 (70.78) | 1,924 (57.55) |  |  |
| Yes | 118 (25.27) | 497 (29.22) | 1,419 (42.45) |  |  |
| Social participant, n(%) |  |  |  | χ²=87.64 | **<.001** |
| Poor | 434 (92.93) | 1,497 (88.01) | 2,669 (79.84) |  |  |
| Good | 33 (7.07) | 204 (11.99) | 674 (20.16) |  |  |
| Method for cooking, n(%) |  |  |  | χ²=99.24 | **<.001** |
| Nonclean energy | 349 (74.73) | 1,146 (67.37) | 1,877 (56.15) |  |  |
| Clean energy | 118 (25.27) | 555 (32.63) | 1,466 (43.85) |  |  |
| Kitchen's ventilation status, n(%) |  |  |  | χ²=154.98 | **<.001** |
| No | 107 (22.91) | 166 (9.76) | 199 (5.95) |  |  |
| Yes | 360 (77.09) | 1,535 (90.24) | 3,144 (94.05) |  |  |
| Mold exposure, n(%) |  |  |  | χ²=80.78 | **<.001** |
| No | 343 (73.45) | 1,487 (87.42) | 2,955 (88.39) |  |  |
| Yes | 124 (26.55) | 214 (12.58) | 388 (11.61) |  |  |
| Distance of residence from the main road, n(%) |  |  |  | χ²=2.15 | 0.342 |
| <200 meters | 203 (43.47) | 714 (41.98) | 1,353 (40.47) |  |  |
| ≥200 meters | 264 (56.53) | 987 (58.03) | 1,990 (59.53) |  |  |
| CO, M (Q₁, Q₃) | 0.88 (0.82,0.93) | 0.88 (0.82,0.95) | 0.85 (0.81,0.93) | χ²=69.63# | **<.001** |
| NO_2_, M (Q₁, Q₃) | 29.91 (18.95,34.36) | 34.04 (19.51,34.36) | 25.27 (18.95,34.27) | χ²=64.49# | **<.001** |
| O_3_, M (Q₁, Q₃) | 104.21 (89.08,109.73) | 104.21 (91.79,108.57) | 92.85 (89.08,108.57) | χ²=150.37# | **<.001** |
| PM_2.5_, M (Q₁, Q₃) | 43.85 (30.07,49.60) | 44.35 (30.07,49.60) | 34.89 (29.90,46.22) | χ²=176.36# | **<.001** |
| PM_10_, M (Q₁, Q₃) | 72.99 (48.90,95.55) | 78.85 (54.13,95.55) | 56.51 (48.13,78.85) | χ²=154.71# | **<.001** |
| SO_2_, M (Q₁, Q₃) | 12.32 (11.73,14.61) | 12.32 (11.06,14.61) | 11.73 (10.67,12.35) | χ²=152.73# | **<.001** |

#: Kruskal-waills test, χ²: Chi-square test, **-:** Fisher exact, ***:** Simulated p-value

Supplementary Table 4. Basic characteristics of research participants in the external validation dataset by frailty (n=718).

| **Variables** | **Total**  **(n = 718)** | **Normal**  **(n = 542)** | **Frailty**  **(n = 176)** | **Statistic** | ***P*** |
| --- | --- | --- | --- | --- | --- |
|  |  |  |  |  |  |
| Age, M (Q₁, Q₃) | 82.00 (74.00, 92.00) | 79.00 (72.00, 87.00) | 93.00 (86.75, 100.00) | Z=-12.29 | **<.001** |
| Gender, n(%) |  |  |  | χ²=13.49 | **<.001** |
| Female | 383 (53.34) | 268 (49.45) | 115 (65.34) |  |  |
| Male | 335 (46.66) | 274 (50.55) | 61 (34.66) |  |  |
| Education, n(%) |  |  |  | χ²=41.66 | **<.001** |
| 0 years | 325 (45.26) | 209 (38.56) | 116 (65.91) |  |  |
| ≤6 years | 269 (37.47) | 223 (41.14) | 46 (26.14) |  |  |
| >6 years | 124 (17.27) | 110 (20.30) | 14 (7.955) |  |  |
| Marital status, n(%) |  |  |  | χ²=54.99 | **<.001** |
| Other | 385 (53.62) | 248 (45.76) | 137 (77.84) |  |  |
| Married | 333 (46.38) | 294 (54.24) | 39 (22.16) |  |  |
| Residence, n(%) |  |  |  | χ²=2.73 | 0.098 |
| Rural | 316 (44.01) | 248 (45.76) | 68 (38.64) |  |  |
| City | 402 (55.99) | 294 (54.24) | 108 (61.36) |  |  |
| Smoking, n(%) |  |  |  | χ²=1.64 | 0.200 |
| No | 602 (83.84) | 449 (82.84) | 153 (86.93) |  |  |
| Yes | 116 (16.16) | 93 (17.16) | 23 (13.07) |  |  |
| Drinking, n(%) |  |  |  | χ²=5.40 | **0.020** |
| No | 600 (83.57) | 443 (81.73) | 157 (89.20) |  |  |
| Yes | 118 (16.43) | 99 (18.27) | 19 (10.80) |  |  |
| Exercise, n(%) |  |  |  | χ²=48.35 | **<.001** |
| No | 459 (63.93) | 308 (56.83) | 151 (85.80) |  |  |
| Yes | 259 (36.07) | 234 (43.17) | 25 (14.20) |  |  |
| Social participant, n(%) |  |  |  | χ²=26.73 | **<.001** |
| Poor | 605 (84.26) | 435 (80.26) | 170 (96.59) |  |  |
| Good | 113 (15.74) | 107 (19.74) | 6 (3.41) |  |  |
| Method for cooking, n(%) |  |  |  | χ²=0.91 | 0.340 |
| Nonclean energy | 464 (64.62) | 345 (63.65) | 119 (67.61) |  |  |
| Clean fuel | 254 (35.38) | 197 (36.35) | 57 (32.39) |  |  |
| Kitchen's ventilation status, n(%) |  |  |  | χ²=0.53 | 0.467 |
| No | 45 (6.27) | 36 (6.64) | 9 (5.11) |  |  |
| Yes | 673 (93.73) | 506 (93.36) | 167 (94.89) |  |  |
| Spring ventilation frequency, n(%) |  |  |  | χ²=1.93 | 0.382 |
| 0 time/week | 25 (3.48) | 21 (3.88) | 4 (2.27) |  |  |
| 1-5 times/week | 253 (35.24) | 185 (34.13) | 68 (38.64) |  |  |
| > 5 times/week | 440 (61.28) | 336 (61.99) | 104 (59.09) |  |  |
| Summer ventilation frequency, n(%) |  |  |  | χ²=0.41 | 0.813 |
| 0 time/week | 16 (2.23) | 12 (2.21) | 4 (2.27) |  |  |
| 1-5 times/week | 151 (21.03) | 111 (20.48) | 40 (22.73) |  |  |
| > 5 times/week | 551 (76.74) | 419 (77.31) | 132 (75.00) |  |  |
| Autumn ventilation frequency, n(%) |  |  |  | χ²=6.14 | **0.046** |
| 0 time/week | 27 (3.76) | 24 (4.43) | 3 (1.71) |  |  |
| 1-5 times/week | 263 (36.63) | 187 (34.50) | 76 (43.18) |  |  |
| > 5 times/week | 428 (59.61) | 331 (61.07) | 97 (55.11) |  |  |
| Winter ventilation frequency, n(%) |  |  |  | χ²=3.51 | 0.173 |
| 0 time/week | 142 (19.78) | 112 (20.66) | 30 (17.05) |  |  |
| 1-5 times/week | 284 (39.55) | 204 (37.64) | 80 (45.46) |  |  |
| > 5 times/week | 292 (40.67) | 226 (41.70) | 66 (37.50) |  |  |
| Overall ventilation index, n(%) |  |  |  | χ²=1.00 | 0.608 |
| 0-3 (low) | 60 (8.36) | 44 (8.12) | 16 (9.09) |  |  |
| 4-5 (intermediate) | 215 (29.94) | 158 (29.15) | 57 (32.39) |  |  |
| 6-8 (high) | 443 (61.70) | 340 (62.73) | 103 (58.52) |  |  |

**Z:** Mann-Whitney test, **χ²:** Chi-square test

**M:** Median, **Q₁:** 1st Quartile, **Q₃:** 3st Quartile

**Supplementary Table 5.** The association between indoor ventilation frequency and frailty (categorical) based on an external validation dataset

|  | **Low frequency** | **Intermediate frequency** | **High frequency** |
| --- | --- | --- | --- |
|  | **OR (95% CI )** | | |
| **Model 1** | Ref. | 0.992 (0.519, 0.999)** | 0.833 (0.451, 0.961)*** |
| **Model 2** | Ref. | 0.957 (0.477, 0.970)* | 0.924 (0.478, 0.956)* |
| **Model 3** | Ref. | 0.975 (0.485, 0.993)* | 0.952 (0.491, 0.997)* |

**Note:** CI is confidence interval; *:p<0.05; **:p<0.01; ***:p<0.001

**Model 1:** Crude model.

**Model 2:** Adjusted for age, gender, education, marital status, and residence.

**Model 3:** Adjusted for model 2 + smoking, drinking, exercise, and social participation.

**Supplementary Table 6.** The association between indoor ventilation frequency and frailty (continuous) based on an external validation dataset.

|  | **Low frequency** | **Intermediate frequency** | **High frequency** |
| --- | --- | --- | --- |
|  | **OR (95% CI )** | | |
| **Model 1** | Ref. | -0.03 (-0.05, -0.01)** | -0.03 (-0.05, -0.02)*** |
| **Model 2** | Ref. | -0.02 (-0.05, -0.01)*** | -0.02 (-0.04, -0.00)*** |
| **Model 3** | Ref. | -0.03 (-0.04, -0.02)*** | -0.02 (-0.05, -0.01)* |

**Note:** CI is confidence interval; *:p<0.05; **:p<0.01; ***:p<0.001

**Model 1:** Crude model.

**Model 2:** Adjusted for age, gender, education, marital status, and residence.

**Model 3:** Adjusted for model 2 + smoking, drinking, exercise, and social participation.

**Supplementary Table 7.** The association between seasonal indoor ventilation frequency and frailty (categorical)

| **Season^a^** | **Low frequency** | **Intermediate frequency** | **High frequency** |
| --- | --- | --- | --- |
|  | **OR (95% CI )** | | |
| **Spring** | Ref. | 0.744 (0.536, 1.032) | 0.897 (0.648, 1.241) |
| **Summer** | Ref. | 0.822 (0.543, 1.245) | 0.809 (0.545, 0.953)* |
| **Autumn** | Ref. | 0.838 (0.592, 1.186) | 0.887 (0.628, 0.922)* |
| **Winter** | Ref. | 0.837 (0.695, 0.887)** | 0.835 (0.715, 0.975)* |

**Note:** OR is odds ratio; CI is confidence interval; *:p<0.05; **:p<0.01

**Fully adjusted model:** Adjusted for age, gender, education, marital status, residence, living arrangement, smoking, drinking, exercise, social participation, methods for cooking, kitchen’s ventilation status, mold exposure, distance of residence from the main road, concentrations of CO, NO_2_ O_3_, PM_2.5_, PM_10_, and SO_2_.

**Supplementary Table 8.** The association between seasonal indoor ventilation frequency and frailty (continuous) based on an external validation dataset.

| **Season^a^** | **Low frequency** | **Intermediate frequency** | **High frequency** |
| --- | --- | --- | --- |
|  | **Beta coefficients (95% CI )** | | |
| **Spring** | Ref. | -0.02(-0.03, -0.01)* | -0.01 (-0.02, -0.01) |
| **Summer** | Ref. | -0.02(-0.04, 0.00) | -0.02 (-0.03, 0.00) |
| **Autumn** | Ref. | -0.01 (-0.03, -0.00)* | -0.01(-0.03, 0.01)* |
| **Winter** | Ref. | -0.01(-0.02, -0.01)* | -0.00 (-0.01, 0.01) |

**Note:** CI is confidence interval; *:p<0.05

**Fully adjusted model:** Adjusted for age, gender, education, marital status, residence, smoking, drinking, exercise, and social participation.
